# Fishing with bed nets on Lake Tanganyika: a randomized survey

**DOI:** 10.1186/1475-2875-13-395

**Published:** 2014-10-07

**Authors:** Kate A McLean, Aisha Byanaku, Augustine Kubikonse, Vincent Tshowe, Said Katensi, Amy G Lehman

**Affiliations:** Lake Tanganyika Floating Health Clinic, Kigoma, Tanzania; The Green World Vision, Kigoma, Tanzania

**Keywords:** Malaria, Insecticide-treated nets, Lake Tanganyika, Bed net misuse, Fishing, Ecology, Fisheries management

## Abstract

**Background:**

Malaria is among the most common causes of death along Lake Tanganyika, a problem which many aid organizations have attempted to combat through the distribution of free mosquito bed nets to high-risk communities. The Lake Tanganyika Floating Health Clinic (LTFHC), a health-based non-governmental organization (NGO), has observed residents of the Lake Tanganyika basin using bed nets to fish small fry near the shoreline, despite a series of laws that prohibit bed net use and other fine-gauge nets for fishing, implemented to protect the near-shore fish ecology. The LTFHC sought to quantify the sources of bed nets and whether they were being used for fishing.

**Methods:**

The LTFHC conducted a survey of seven lakeside villages in Lagosa Ward, Tanzania. The government has divided each village into two to six pre-existing geographic sub-villages depending on population size. Seven households per sub-village were chosen at random for survey administration. The survey consisted of 23 questions regarding mosquito bed net practices, including the use of bed nets for fishing, as well as questions pertaining to any perceived changes to the fish supply.

**Results:**

A total of 196 surveys were administered over a four-week period with a 100% response rate. Over 87% of households surveyed have used a mosquito bed net for fishing at some point. The majority of respondents reported receiving their bed net for free (96.4%), observing “many” residents of their village using bed nets for fishing (97.4%), and noticing a subjective decrease in the fish supply over time (64.9%).

**Conclusions:**

The findings of this study raise concerns that the use of free malaria bed nets for fishing is widespread along Lake Tanganyika, and that this dynamic will have an adverse effect on fish ecology. Further studies are indicated to fully define the scope of bed net misuse and the effects of alternative vector control strategies in water-based communities.

**Electronic supplementary material:**

The online version of this article (doi:10.1186/1475-2875-13-395) contains supplementary material, which is available to authorized users.

## Background

Malaria is one of the most significant health problems in the world, with 207 million cases and 627,000 deaths per year, the majority of which occur in sub-Saharan Africa [1]. The use of insecticide-treated nets and long-lasting, insecticide-treated nets (ITNs and LLINs) can reduce malaria morbidity and mortality, particularly among children and pregnant women [2, 3]. While these nets are intended by humanitarian donors to be used over sleeping spaces, there have been a number of reports of misuse of nets for fishing [4–6], to make wedding dresses and veils [7] and to cover crops [8]. There is very little in the scientific literature addressing these trends, and what has been published suggests that misuse has not been adequately quantified and may even be a trivial problem [9].

The LTFHC has a full-time team on the shores of Lake Tanganyika, placing staff members in unique position to observe patterns of bed net use in lakeside communities where malaria is endemic [10, 11]. Lake Tanganyika is the second largest freshwater lake in the world, with an area of 32,900 sq km, and a depth of 1,433 m [12]. Similar to the rest of Tanzania, the most common vector in the area is *Anopheles gambiae* [13], and although there have been studies in Tanzania suggesting *An. gambiae* resistance to insecticides [14], there has not been confirmation of this specifically along Lake Tanganyika. ITN distribution in Tanzania began in 2004–2005, with LLINs becoming more universally used by 2011 [15]. The Tanzanian Government has developed a subsidy/voucher scheme, with rates of LLIN use reported to be up to 75% in some regions as of 2011 [16]. Thus, it is unsurprising that bed nets are the primary means of vector control along the Lake. However, the rates of malaria prevalence among children in Kigoma Region, which is along Lake Tanganyika, remain the second highest in Tanzania at 26% [10]. This is unchanged since 2007 [17], suggesting that bed net distribution has not had the intended effect.

**Figure 1:**
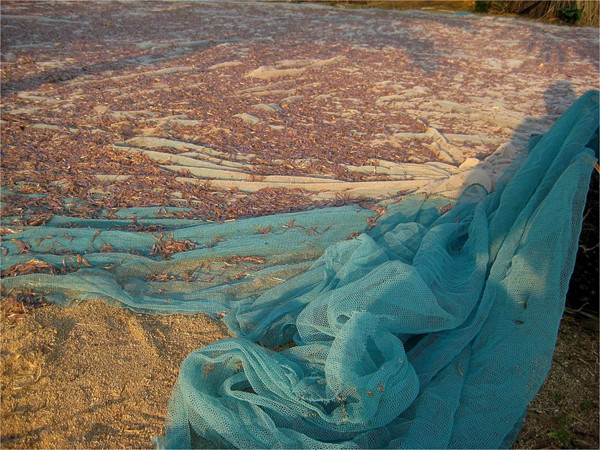
**Bed nets used for fishing along Lake Tanganyika.**

**Figure 2:**
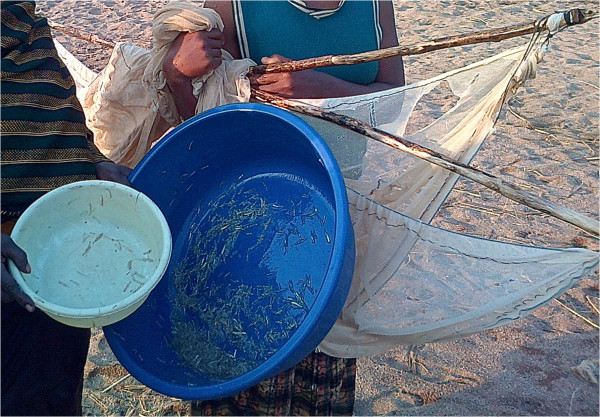
**Fish fry caught in Lake Tanganyika with a bed net.**

The average annual income in Tanzania is US$630, with the majority of the poor living in rural areas such as those along Lake Tanganyika [18]. These communities primarily subsist on fishing for *Stolothrissa tanganicae* and *Luciolates stappersii*, which typically range from 6.4 to 21.8 cm when caught [19]. Annually, over 200,000 tons of fish are harvested from Lake Tanganyika [20] and fishing nets are usually obtained from commercial sources and cost around US$25. Tanzanian law currently prohibits the use of bed nets and other fine-gauge nets for fishing given the potential for harm to the fish supply through removal of fish fry [21]. There is growing concern among experts regarding increased illegal selling of these fish fry and the effect this could have on Lake Tanganyika’s fish supply [22]. Additionally, Article 7 of the Convention on Sustainable Management of Lake Tanganyika, signed by all four riparian countries surrounding the Lake, directs the contracting states to establish a framework fisheries management plan. This management plan is intended to develop and implement harmonized national fisheries policies and regulations, and to promote community participation in fisheries management. Despite this, LTFHC noted multiple villages in Tanzania and the Democratic Republic of Congo using bed nets to fish (Figures 1 and 2), leading the team to hypothesize that this is a common practice along Lake Tanganyika. Exploration of this hypothesis has far-reaching implications, given that alternative bed net use has also been noted outside of sub-Saharan Africa [23].

## Methods

The LTFHC conducted a randomized survey of seven villages in Lagosa Ward (Additional file 1) in western Tanzania along Lake Tanganyika: Igalula, Buhingu, Rukoma, Kalilani, Mgambazi, Katumbi, and Nkonkwa (Additional file 2). This region was chosen due to logistic feasibility, but also because the LTFHC has a partnership with the Green World Vision, whose staff are well known in this area. The team believed having the survey carried out by people who could easily foster community trust and openness through commonality of tribe and language would lead to the most thorough results. According to local officials, these villages have a total population of 40,805. The government has divided each village into two to six pre-existing geographic sub-villages, depending on population size. We estimated a sample size of at least 150 based on the total population given above, a 95% confidence level, an 8% margin of error, and a response distribution of 50% for the proportion of households using bed nets for fishing. Seven households per sub-village were therefore randomly chosen, through the Random Walk Method [24] for survey administration by the field team. The survey consisted of 23 questions regarding mosquito bed net practices, including the use of bed nets for fishing, as well as questions regarding the participants’ perception of any changes to the fish supply. (Examples of survey questions are as follows: Do you ever use a bed net for fishing? If you use a bed net for fishing, how often do you do so? Have you ever seen anyone else using a bed net for fishing? If so, how many people have you seen? Have you noticed any changes to the fish supply in Lake Tanganyika over time?) Surveys were written and administered by native speakers in Swahili, which is the most commonly spoken language in the region. No compensation was given for participation and it was made clear that participation was completely voluntary. The Green World Vision is a registered NGO in Tanzania and has governmental approval to conduct research related to natural resources management, community development and ecology. Statistical analysis was performed using STATA 13.1 (College Station, Texas, USA). In order to examine whether poverty predisposed using a bed net to fish, the number of children in a household was used as a proxy for socio-economic status. This decision was based on a previous body of work suggesting negative economic consequences are linked to high birth rates [25–27]. Logistic regression was performed to explore this association.

## Results

A total of 196 surveys were administered over a four-week period in November 2013, with a 100% response rate. This high response rate was attributed to the fact that those performing the survey were well known to and trusted by the community. On average, each household surveyed had 5.7 members (95% CI 5.4-6.0), with 3.3 children (95% CI 3.0-3.5) (Table 1). All households reported owning a bed net at some point in time, with the median ownership duration of six to 12 months (minimum less than six months, maximum over five years). The majority (189 (96.4%, 95% CI 92.5-98.4%)) reported obtaining that bed net from an NGO at no cost. Those who paid for their bed nets spent an average of 5,100 TSH (95% CI 4,307-5,818 TSH, approximately US$3). A total of 171 households (87.2%, 95% CI 83.6-90.3%) (Table 2) reported having ever used a bed net to fish, and 107 (62.6% of those fishing with a bed net, 95% CI 52.8-67.4%) reported doing so more than once per day, over a previous period averaging six to 12 months.

**Table 1:** **Survey population demographics**

Household characteristics	Mean (n = 196)
Age of respondent	34.2 (95% CI 31.6-36.9)
Occupation: fishing	13 (6.6%)
Occupation: farming	52 (26.5%)
Occupation: fishing and farming	131 (66.8%)
Number of people in household	5.7 (95% CI 5.4-6.0)
Number of children	3.3 (95% CI 3.0-3.5)
Number of children under age 5	1.2 (95% CI 1.1-1.3)

**Table 2:** **Percentage of households that have fished with bed nets, by village**

Village name	Number surveyed	Fish with bed nets (%)
Igalula	35	31 (88.6%)
Buhingu	35	29 (82.9%)
Rukoma	28	25 (89.3%)
Kalilani	28	24 (85.7%)
Mgambazi	28	25 (89.3%)
Katumbi	28	24 (85.7%)
Nkonkwa	14	13 (92.9%)
**Total**	**196**	**171 (87.2%)**

All but one respondent (170/171, 99.4% of those fishing with a bed net, 95% CI 98.3-100.0%) reported using the bed net to catch fish fry at the lakeshore, as opposed to using it for offshore fishing from a boat. The most frequent reason (46.8%, 95% CI 38.5-53.5%) given for fishing with bed nets was needing a fishing net to generate income but being unable to afford one, with hunger as the second most reported reason (38.0%, 95% CI 29.5-44.0%). Respondents were near unanimous (191 (97.4%, 95% CI 95.3-99.7%)) in reporting seeing “many” others in their village use bed nets to fish. Most of those surveyed (170 (86.7%, 95% CI 82.0-91.6%)) admitted awareness that fishing with bed nets is illegal in Tanzania. Although a trend was seen in this direction, logistic regression did not show a statistically significant association between having a higher number of children (and therefore more people to feed) and fishing with bed nets (p = 0.069). One-hundred and eleven households (64.9% of those fishing with a bed net, 95% CI 56.9-71.1%) reported a subjective decrease in the fish supply in Lake Tanganyika.

## Discussion

This study suggests that the use of bed nets for fishing is a widespread, frequent and commonly accepted practice along Lake Tanganyika. This observation raises concerns that this practice may also be occurring in other waterside communities in sub-Saharan Africa. A previous study has suggested similar problems on Lake Victoria [28], however no large-scale studies have been published to date on this issue. The LTFHC is troubled by the revelations of this study for the following reasons: not only does the use of bed nets for fishing suggest they are not being used for the intended purpose of malaria prevention, but this practice may actively be harmful in other ways. For example, 12 million residents in the Lake Tanganyika basin depend on fish as their primary source of dietary protein [29]. Fishing with fine-gauge nets is already outlawed in Tanzania for fear that removal of fish fry will decimate these important fish populations. In addition, insecticides used to treat bed nets, such as permethrin, are known to be toxic to aquatic life [30]. Permethrin is moderately soluble in water, creating the possibility that fishing with bed nets could lead to leaching of this chemical into the lake, and subsequent damage to surrounding fish. Lake Tanganyika is home to over 300 fish species, two-thirds of which are endemic [19], making this a potential danger to biodiversity. Permethrin is also classified by the Environmental Protection Agency as “likely to be carcinogenic to humans” [30], which poses a threat to residents in the villages surveyed, as they depend on the Lake for their drinking water.

There are likely complex motivating factors related to how bed nets are used within households. Previous research has shown that the poorest levels of society have the highest rates of parasitaemia in the region and are also the least likely to sleep under bed nets [31], however these same families are also battling other serious concerns, such as hunger. Economic pressures are known to impact malaria prevention [32], so it is certainly possible that behavioural change will not occur until either those pressures decrease (i.e., food security improves and hunger is less prevalent) or those pressures are disengaged from bed net use. This could mean changing standard vector control from bed nets, which are easy to mobilize for other uses, to something stationary, such as wall treatments or newly developed, individual spatial repellents.

## Conclusions

When considered as a whole, examining bed net use for fishing sheds light on a range of issues: malaria mortality, food security, biodiversity, and preservation of safe water sources. As such, it is crucial for the international community to explore this issue further. More rigorous studies of the effects of fishing with bed nets on the fish supply and water quality are needed, as well as investigations of alternative personal vector control methods. NGO bed net distributors should also make every effort to assure proper bed net use. Additional community education regarding appropriate bed net use and the dangers of using those nets for fishing may be worth considering, as is the organization of behavioural change communication campaigns, which have been shown to be effective in addressing other public health problems [33]. The LTFHC conducted a widespread bed net distribution along Lake Tanganyika in the Democratic Republic of Congo with concurrent education and specific discussion of the laws prohibiting fishing. When staff returned to all health areas that received nets, they found bed net fishing in only a single village. These staff members personally confiscated the nets used for fishing, as the LTFHC does not wish to facilitate illegal activity. The effectiveness of such a strategy could also be studied going forward.

## Electronic supplementary material

Additional file 1: **Map 1 Lagosa Ward and Lake Tanganyika.** (JPEG 218 KB)

Additional file 2: **Map 2 Villages surveyed.** (JPEG 86 KB)
