# Supplementary figures and images for: Fishing with bed nets on Lake Tanganyika: a randomized survey
Source: Malar J. 2014 Oct 7;13:395. doi: 10.1186/1475-2875-13-395 (PMC4198669; doi:10.1186/1475-2875-13-395)

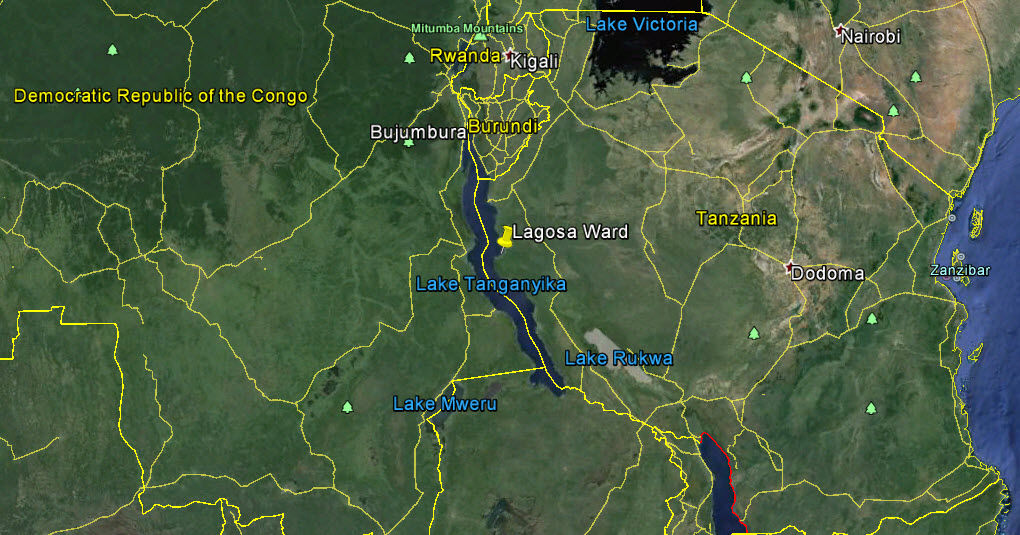

Supplement: Supplementary file 1 — Additional file 1: Map 1 Lagosa Ward and Lake Tanganyika. (JPEG 218 KB) [file 12936_2014_3557_MOESM1_ESM.jpeg]

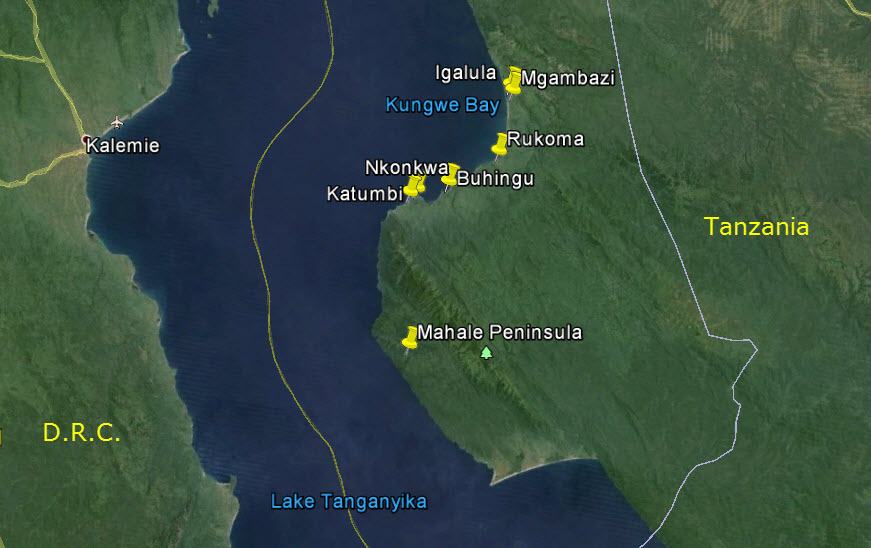

Supplement: Supplementary file 2 — Additional file 2: Map 2 Villages surveyed. (JPEG 86 KB) [file 12936_2014_3557_MOESM2_ESM.jpeg]
